# Genome-Wide Association Studies of Agronomic and Yield Traits in Sweet Corn (*Zea mays* L. var. *saccharata*)

**DOI:** 10.3390/plants15152406

**Published:** 2026-08-06

**Authors:** Yanchao Du, Jingwen Xu, Huiming Li, Mingxing Zhou, Xu Pang, Jianbing Yan, Ye He, Guowu Lian, Faqiang Feng

**Affiliations:** 1 Shanxi Key Laboratory for Germplasm Innovation and Genetic Improvement in Staple Crop, Sorghum Research Institute, Shanxi Agricultural University, Jinzhong 030600, China; duyanchao@sxau.edu.cn (Y.D.);; 2 Guangdong Provincial Key Laboratory of Plant Molecular Breeding, South China Agricultural University, Guangzhou 510642, China; xu030314@163.com (J.X.);; 3 Shanxi Province Agricultural Technology Extension Service Center, Taiyuan 030106, China

**Keywords:** sweet corn, genome-wide association study, plant architecture, ear traits, candidate genes, marker–trait associations

## Abstract

Sweet corn is a globally important dual-purpose crop for both food and fresh vegetables. The plant architecture and ear-related traits directly determine its yield potential and field ecological adaptability. To elucidate the genetic architecture of these traits and identify superior alleles for breeding, we conducted a genome-wide association study (GWAS) on 11 agronomic traits using 30,597 high-quality SNP markers in a panel of 101 elite sweet corn inbred lines. Population genetic structure was analyzed using sparse non-negative matrix factorization (sNMF) and discriminant analysis of principal components (DAPC) algorithms, revealing three main clusters and six subpopulations. The clustering pattern was highly consistent with germplasm origin. Association mapping with the fixed and random Circulating Probability Unification (FarmCPU) model identified 16 significant marker–trait associations (MTAs), distributed across seven target agronomic traits. The phenotypic variance explained (PVE) by individual loci ranged from 8.0% to 16.0%. Among these, five stable MTAs across environments, a novel ERN locus (SNP25518) specific to sweet corn, and most association intervals overlapped with previously reported quantitative trait loci (QTLs). Within the ±0.15 Mb (defined by LD decay) flanking windows around the significant SNP loci, a total of 236 candidate genes were annotated, which are primarily involved in hormone signaling, carbon and nitrogen metabolism, cell division, and plant growth and development. In summary, this study dissected the genetic basis of key agronomic traits in sweet corn and provides a foundation for marker-assisted selection and functional validation.

## 1. Introduction

Sweet corn (*Zea mays* L. var. *saccharata*) is an important vegetable crop prized for its high soluble sugar content, low starch concentration, unique flavor, and rich nutritional profile including carotenoids. Global demand continues to grow as it is widely consumed both fresh and processed [1,2]. The crop adapts to diverse climatic conditions and is cultivated worldwide, with a notable increase in commercial production in recent years [3]. In China, sweet corn is an economically valuable crop grown on a large scale in tropical and subtropical regions to meet rising market needs [4]. Plant architecture and yield-related agronomic traits play a decisive role in achieving high and stable yields. Structural characteristics such as plant height and ear height directly affect canopy light interception efficiency and density tolerance, making them fundamental for optimizing planting density and maximizing yield [5,6]. Yield components including fresh ear weight, ear length, and ear diameter exhibit significant genotype-by-environment interactions [3,7]. Therefore, systematically dissecting the genetic basis of key agronomic traits is essential for breeding high-yielding and stress-tolerant sweet corn varieties.

Agronomic traits in maize are complex quantitative traits whose phenotypic variation is governed by polygenic interactions and environmental factors [6,8]. For plant architecture, numerous QTLs have been mapped, and a few causal genes have been cloned [9,10,11,12]. Zhao et al. [13] conducted a GWAS using 160 maize inbred lines and identified 9, 23, 18, 8, and 32 loci significantly associated with tassel primary branch number, tassel length, ear height, plant height, and the ear height-to-plant height ratio, respectively. Using a recombinant inbred line (RIL) population derived from Ye478 × Qi319, Shang et al. [14] detected 46 QTLs for plant height, ear height, and stem diameter, each explaining 3.5% to 17.7% of the phenotypic variance across environments. For leaf number above the ear, map-based cloning successfully identified the key gene *ZmTE1* underlying a major QTL, which regulates leaf development and source–sink relationships and plays a critical role in grain filling [11]. Known functional genes such as *zfl2* have been confirmed to be significantly associated with tassel length, while *na1*, *td1*, and *d3* have been reported to be involved in plant height regulatory networks [15]. However, sweet corn has undergone strong selection for flavor and eating quality, leading to a distinct genetic architecture for agronomic and yield traits compared to common maize. Reports on stable major QTLs for stem diameter and tassel length in sweet corn remain limited, and most studies have been conducted in common maize or mixed germplasm. High-resolution mapping and gene cloning specifically for sweet corn are still insufficient. This knowledge gap is specifically addressed in our study by performing GWAS in an elite sweet corn panel.

Ear traits are core determinants of yield in sweet corn, and their genetic dissection is critical for high-yield breeding. In terms of QTL mapping, numerous studies using recombinant inbred line (RIL), doubled haploid (DH), and GWAS populations have systematically identified a large number of QTLs, with a few genes cloned as well [12,16]. Using 209 RIL families, QTL mapping of ten ear-related traits revealed that individual QTLs explained 0.40–36.76% of the phenotypic variance. In the IBM Syn10 population, combined linkage and association analyses detected 100 QTLs and 138 significant SNPs, and the candidate gene *GRMZM2G098557* regulating kernel row number was validated through segregation analysis [16]. To date, the cloned genes related to ear number in maize include *KRN1* [17], *KRN2* [18], and *KRN4* [19]. For ear diameter, a meta-*QTL* analysis integrating 760 QTLs from 37 studies identified 19 meta-*QTL* (MQTL) intervals stably associated with ear traits [20]. Using multi-parent RIL populations combined with QTL and GWAS approaches, key loci *qE1-1*, *qE1-5*, and *qE1-7* as well as five candidate genes were identified [21,22]. *EAD1* (*Ear Apical Degeneration 1*), cloned via map-based cloning, was shown to regulate ear tip meristem development, and its mutation significantly reduced ear length [23]. Meanwhile, *GRMZM2G098557* was functionally validated as a key regulator of KRN [24]. Furthermore, transcriptome and interaction network analyses identified candidate genes associated with ear diameter, such as *Zm00001d005740* (bHLH-transcription factor 138) and *Zm00001d051328* (WRKY-transcription factor 98) [25,26]. Notably, QTLs for ear traits exhibit significant chromosomal hotspot regions, and some loci are pleiotropic [16,27]. However, major QTL mapping and gene cloning for ear-related traits in sweet corn germplasm remain scarce.

GWAS, leveraging high-throughput molecular markers and the efficient use of natural population diversity, has become a core strategy for dissecting the genetic basis of complex quantitative traits in maize [28,29]. In sweet corn, GWAS has been applied to dissect traits such as plant morphology and grain quality. Dang et al. [6] used a panel of 190 sweet corn and 287 waxy corn inbred lines and identified 184, 45, and 68 significant SNP loci associated with plant height, ear height, and tassel branch number, respectively. Chen et al. [30] detected 92 significant SNP loci associated with plant height and other traits in a mixed population of 188 sweet corn, waxy corn, and hybrid lines. For grain quality, GWAS has been applied to map genetic loci for key nutritional components such as carotenoids [31], tocopherols [32], and folates [33]. Qu et al. [34] identified 49 significant SNPs associated with 100-kernel weight, kernel length, kernel width, and kernel diameter in a mixed population of 447 waxy and sweet corn lines. Thus, a multi-trait joint analysis strategy is gradually being adopted in sweet corn research.

However, current multi-trait GWAS studies in sweet corn still face challenges. Most studies have used mixed populations containing sweet corn, whereas fewer have utilized elite sweet corn inbred line breeding panels. Here, we employed the FarmCPU model, which iteratively uses fixed and random effects to improve detection power while controlling false positives, outperforming traditional mixed linear models. The objective of this study was to conduct a genome-wide association analysis of plant architecture and ear traits using a panel of 101 elite sweet corn inbred lines developed in our laboratory. This study expects to identify novel associated loci and pleiotropic regions underlying agronomic and yield traits in sweet corn, understanding the genetic architecture of agronomic traits in sweet corn and providing solid support for molecular breeding.

## 2. Results

### 2.1. Phenotypic Variation of Agronomic Traits

**Figure 1:**
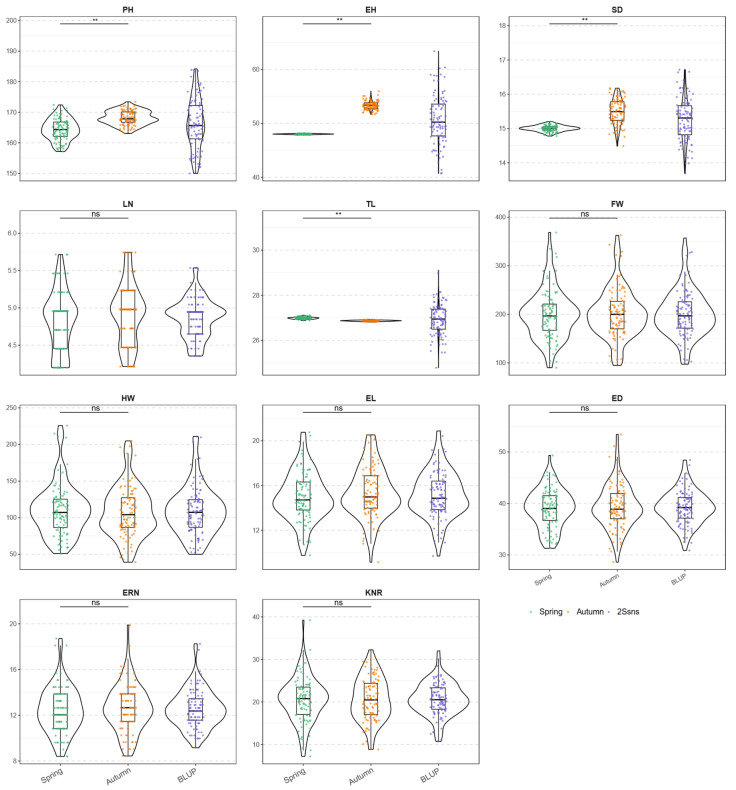
Violin plots of the 11 agronomic traits with the comparison of spring and autumn means. PH, plant height (cm); EH, ear height (cm); SD, stem diameter (mm); LN, leaf number above the ear; TL, tassel length (cm); FW, fresh ear weight (g); HW, husked ear weight (g); EL, ear length (cm); ED, ear diameter (cm); ERN, ear row number; KNR, kernel number per row. ns and ** represents nonsignificance and significance at the 0.01 level, respectively.

Descriptive statistics for 11 agronomic traits in sweet corn are summarized in Table S1 and Figure 1. Best linear unbiased predictions (BLUPs) were calculated for each trait as adjusted means by linear mixed models, based on data from spring and autumn, and their combined analysis (2 Ssns). Phenotypic differentiation was observed across seasons for most traits, indicating notable seasonal environmental effects. The mean plant height (PH) and ear height (EH) across both seasons were 166.27 cm and 50.67 cm, respectively. Higher mean values for PH and EH in autumn than in spring suggested that autumn field conditions were more favorable for vegetative growth, effectively promoting increases in both PH and EH. In contrast, number of leaves above the ear (LN), tassel length (TL) and stem diameter (SD) showed limited seasonal variation, with small differences between spring and autumn. Across both seasons, the mean values for LN, TL, and SD were 4.87 leaves, 26.93 cm, and 15.25 mm, respectively.

Key yield and ear traits, including fresh ear weight (FW), ear row number (ERN), ear length (EL) and ear diameter (ED), exhibited varying degrees of seasonal fluctuation. The mean FW in spring and autumn was 200.31 g and 201.25 g, respectively, indicating a negligible seasonal difference. EL, ED, and LN showed slightly higher mean values in autumn than in spring. The coefficient of variation (CV) differed substantially among traits. Husked ear weight (HW), FW, and KNR displayed relatively high CV values, reaching 30.49%, 23.83%, and 18.64% in the combined analysis, respectively, indicating abundant phenotypic variation in these yield-related traits within the sweet corn panel. In contrast, PH, SD, LN and TL had low CV values, all below 6% in the combined analysis.

Heritability analysis revealed moderate to high heritability for all 11 agronomic traits. Across individual seasons, broad-sense heritability (*H*^2^) estimates ranged from 0.59 to 0.91. In the combined analysis, *H*^2^ increased for most traits. HW, ERN, and KNR exhibited the highest broad-sense heritability values, reaching 0.97, 0.92, and 0.92, respectively, in the combined analysis. TL had the lowest broad-sense heritability, consistently estimated at 0.59 across spring, autumn, and the combined analysis.

Analysis of variance revealed that all 11 traits were typical quantitative traits jointly regulated by genotype and seasonal environment (Table 1). With the exception of TL, genotype effects were highly significant at the level of *p* < 0.01 for the remaining ten traits, indicating reliable and stable genetic differences among the sweet corn population. In terms of season, only PH, EH, SD, and LN exhibited significant differences between spring and autumn. Among these traits, EH showed the largest seasonal mean square of 4281.54, followed by PH, suggesting that vegetative growth traits are more susceptible to environment. Furthermore, significant genotype × season interaction effects were detected for LN, FW, HW, EL, ED, ERN, and KNR, indicating variation in phenotypic plasticity among genotypes for these traits.

### 2.2. Phenotypic Correlations of Agronomic Traits

**Figure 2:**
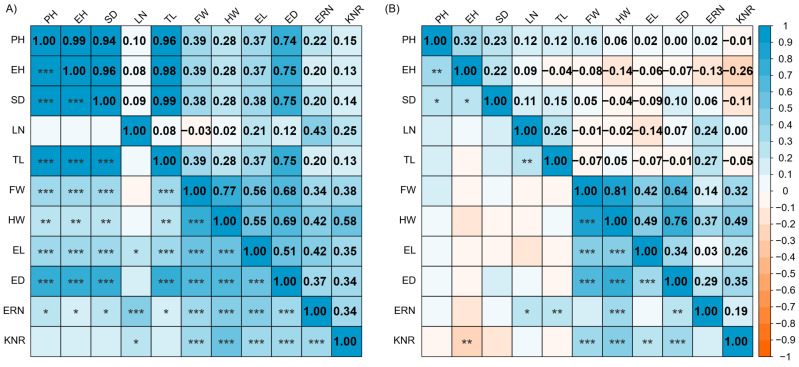
Phenotypic correlations across 11 agronomic traits measured in spring (**A**) and autumn (**B**). Blue denotes positive correlations, while red represents negative correlations. Correlations with *p* > 0.05 (non-significant) are filled in with blanks. *, ** and *** indicate significant correlations at the level of 0.05, 0.01 and 0.001.

Phenotypic correlations among 11 agronomic traits were analyzed for each season (Figure 2). In the spring planting environment, all pairwise correlation values among four plant architecture traits (PH, EH, SD, TL) were highly significant and positive, with the highest coefficients of 0.99, indicating strong co-regulation among these traits in spring. In contrast, LN showed weak correlations with four plant architecture traits and coefficients ranged from 0.08 to 0.10. Most ear-related traits (FW, HW, EL, ED, ERN, KNR) exhibited significant positive correlations with each other. Moreover, FW, HW, EL, and ED were significantly and positively correlated with PH, EH, SD, and TL; ERN was positively correlated with other agronomic traits, while KNR showed nonsignificant correlations with PH, EH, SD and TL. LN were positively correlated with EL, ERN and KNR.

In the autumn planting environment, the correlation patterns among 11 agronomic traits differed from those in spring. PH, EH and SD exhibited weak positive correlations with each other, and TL was positively correlated only with LN. Within ear and yield traits, stable positive correlations were maintained, and these were less affected by seasonal changes. However, the overall associations between plant architecture traits and ear/kernel traits were altered. EH exhibited a significant negative correlation with KNR and ERN were positively correlated with LN and TL.

### 2.3. Population Genetic Structure Analysis

The raw SNP data for 101 sweet corn inbred lines contained 37,747 SNPs. Markers lacking allele information or physical map positions were removed. Duplicated positions were then filtered out. Sites with more than 5% missing data were also discarded. After removing markers with a minor allele frequency (MAF) < 0.05, a total of 30,597 SNPs were retained for downstream analysis, distributed across all ten maize chromosomes (Figure S1).

**Figure 3:**
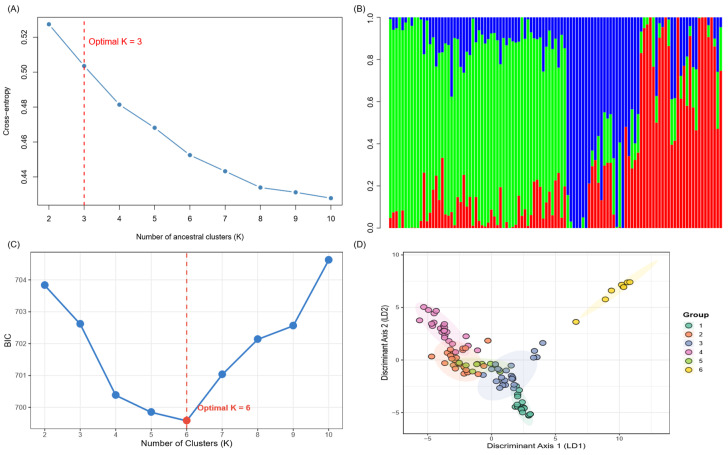
Genetic structure of the studied association panel. (**A**) Most probable number of clusters, defined with LEA method. (**B**) Clusters according to sNMF analysis, each colour represents one ancestral genetic cluster. (**C**) Most probable number of groups for DAPC was defined using Bayesian Information Criterion (BIC) plotted against the number of clusters. (**D**) Grouping of the genotypes in DAPC. Colours correspond to six genetic groups as shown in the legend, and shaded ellipses indicate the 95% confidence intervals for each genetic group.

Two methods were employed to investigate the genetic structure of the association panel. Based on sNMF, the 101 sweet corn elite inbred lines were grouped into three clusters (Table S2; Figure 3A,B). The first cluster (red) contained 23 genotypes, the second (green) and third (blue) comprised 53 and 19 genotypes, respectively, and five genotypes with maximum ancestry coefficient Q < 0.5 were not assigned to any cluster and were considered an admixed group.

DAPC suggested the presence of six groups (Table S2; Figure 3C,D). Group I consisted of 18 genotypes, primarily derived from second-cycle lines of tropical sweet corn varieties collected from southern part of China, including Guangdong, Guangxi, and Yunnan province. These genotypes were concentrated in the third quadrant of the coordinate plot, representing a group relatively distant from others (Figure 3D). Group IV comprised 21 genotypes, mainly from Beijing, Shandong, and Hebei, and these genotypes were concentrated in the second quadrant. Groups II, III, and V consisted of 24, 23, and 7 genotypes, respectively, mainly collected from central China; these genotypes were located near the origin of the coordinate system. Group VI included eight genotypes, all derived from introduced American sweet corn varieties. sNMF reveals coarse genetic groups (three clusters), while DAPC further resolves subpopulations (six groups) due to its higher sensitivity to structure; using both methods provides robust validation of the population structure.

### 2.4. Linkage Disequilibrium

**Figure 4:**
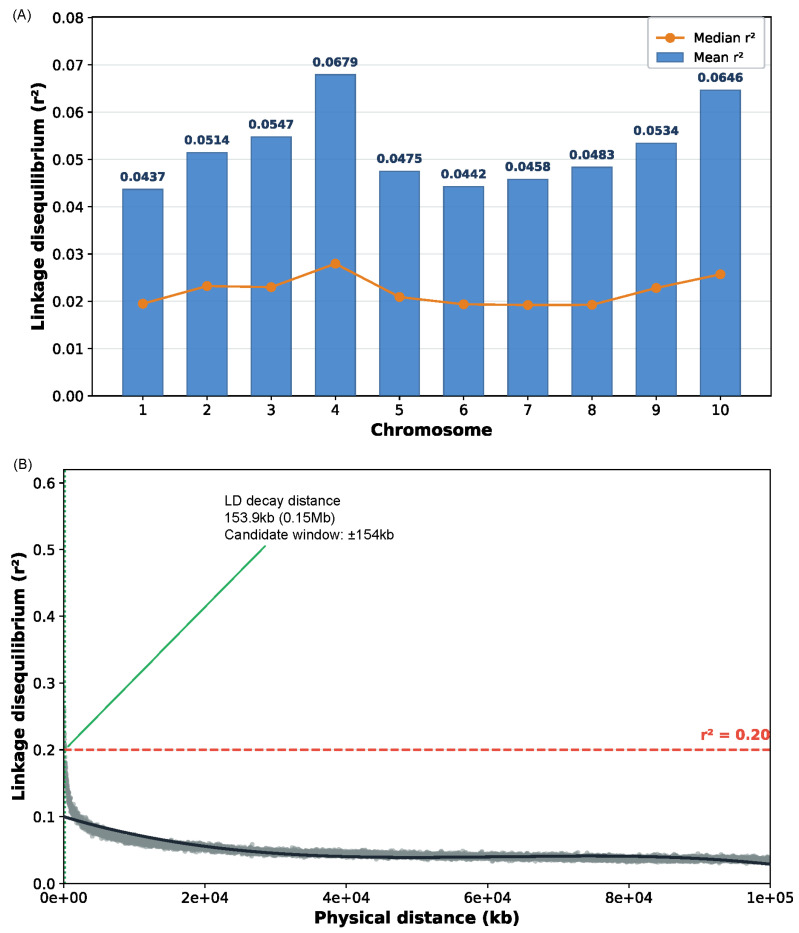
The whole-genome *LD* decay of the association panel. (**A**) Mean (blue bars) and median (orange line) of linkage disequilibrium (*r*^2^) across each of the 10 chromosomes. (**B**) Whole-genome LD decay. The red dotted line depicts *LD* decay as a function of physical distance, while the green dotted line indicates the critical threshold of *r*^2^ = 0.20.

Genome-wide *LD* analyses were performed and revealed that 24.74% of marker pairs were insignificant *LD* (*p* < 0.01), with an average *r*^2^ of 0.051 (Figure 4A). Across individual chromosomes, the mean proportion of marker pairs in *LD* was 25.09%, with an average *r*^2^ of 0.052. Chr 4 exhibited the highest percentage of *LD* (approximately 31.21%), while Chr 1 showed the lowest (21.88%).

In the study population, genome-wide *LD* decayed below the threshold of (*r*^2^ = 0.20) at a distance of 0.15 Mb (Figure 4B). The *LD* decay distance varied among chromosomes, ranging from 0.075 Mb on Chr 1 to 0.215 Mb on Chr 10. The ±0.15 Mb region flanking an SNP associated with a trait was considered the confidence interval for the SNP.

### 2.5. Association Mapping

To identify marker–trait associations, we performed genome-wide association analysis using the FarmCPU model with a Bonferroni-corrected significance threshold of *p* < 3.26 × 10^−5^. A GWAS were conducted for spring and autumn, and 2Ssns (Table 2 and Figure S2). A total of 16 significant MTAs were identified for seven traits: LN, FW, EL, ERN, KNR, SD, and TL. Among these, five SNPs were detected in multiple environments. SNP25518 on Chr 5 was consistently associated with ERN across all three environments. SNP29364, SNP3770, SNP3775, and SNP41368 were located on Chr 7, 1, 1 and 1, respectively, which were detected in two environments; SNP29364 was associated with ERN, while SNPs 3770, 3775, and 41368 were all associated with KNR.

In the spring, ten trait-associated SNPs were identified, belonging to seven target traits (Table 2 and Figure S2). A cluster of closely linked SNPs was detected for FW on Chr 5. For LN, SNP53027 and SNP27309 were located on Chr 6, with −log_10_ (*p*) values of 4.75 and 4.67, respectively, both showing an allelic effect of −0.20 and explaining 11.98% and 12.24% of the phenotypic variance, respectively. For FW, three significant SNPs were mapped on Chr 5. SNP2942 and SNP2943, only 179 bp apart, were considered the same locus, with an effect of 25.6 and a PVE of 11.68%; SNP53698 on Chr 5 was an independent associated locus with a PVE of 11.99%. For EL, SNP48185 was detected on Chr 1, with an effect of −1.75 and a PVE of 8.03%, equivalent to a ~1.75 cm reduction in ear length. For ERN, SNP29364 and SNP25518 explain 10.88% and 10.22% of the PVE, respectively. For KNR, SNP3775 and SNP41368 were detected on Chr 1, representing the most significant associations in the spring environment, both showing negative effects and explaining 12.37% and 11.95% of the PVE, respectively.

In the autumn environment, five significant MTAs were detected, involving four traits: SD, TL, ERN, and KNR (Table 2 and Figure S2). For SD, SNP29274 was detected on Chr 7, with an effect of 0.21 and the highest PVE (15.11%) among autumn-associated loci, which corresponds to a 0.21 cm increase in stem diameter. For TL, SNP25836 was identified on Chr 5, explaining 11.82% of the phenotypic variance. For ERN, two associated SNPs, SNP25518 and SNP47366 on Chr 2, were detected, both with negative effects and PVE values of 9.82% and 12.33%, respectively. For KNR, SNP3770 was identified on Chr 1, with an effect of 2.66 and a PVE of 14.40%, which corresponds to a 2.66 increase in kernel row number.

In the combined 2Ssns environment, seven significant SNP–trait associations were detected, covering LN, ERN, and KNR (Table 2 and Figure S2). For ERN, three significant loci were identified. SNP25518 and SNP29364 were stable MTAs across environments, and a new locus, SNP25505, was detected on Chr 5. SNP25505 is close to SNP25518 with a physical distance of <0.15 Mb and showed a consistent genetic effect, thus they were considered the same locus. All three SNPs exhibited negative allelic effects, with PVE ranging from 10.05% to 12.53%, corresponding to a consistent reduction in ear row number across environments. For KNR, three significant SNPs (SNP3770, SNP3775 and SNP41368) were detected repeatedly across multiple environments. SNP3775 and SNP41368 on Chr 1 are within 0.15 Mb of each other, indicating tight linkage, and were assigned to the same candidate interval. The PVE of these three loci ranged from 9.74% to 16.28%, with SNP3770 showing the highest value. For LN, only SNP2191 was detected on Chr 9, with an effect of 0.13 and a PVE of 11.10%.

### 2.6. Candidate Genes

To identify candidate genes underlying the 11 agronomic traits, we examined the physical genomic positions harboring the MTAs and further searched for high-confidence candidate genes within the ±0.15 Mb interval flanking each peak SNP (the *LD* distance decay to *r*^2^ = 0.20). Among the 16 peak SNPs, 11 SNPs were located within annotated genes, while five located in intergenic regions. The search at the peak SNP positions yielded 15 candidate genes, of which only five had been annotated including *GRMZM2G361164*, *GRMZM2G703466*, *GRMZM2G030275*, *GRMZM2G136975*, and *GRMZM2G116140*. GRMZM2G361164 (polymerase II transcription-mediator 1) regulates RNA polymerase II transcription. GRMZM2G703466 (UPF0187 protein, chloroplastic) is predicted to be involved in plastid development. GRMZM2G030275 (biotin synthase) catalyzes the final step of biotin biosynthesis, essential for fatty acid metabolism and cell division. GRMZM2G136975 (histidinol-phosphate aminotransferase 2, chloroplastic) participates in histidine biosynthesis, providing precursors for nucleotide and hormone metabolism. GRMZM2G116140 (R2R3MYB-domain protein) regulates anthocyanin biosynthesis and cell elongation. Within the ±0.15 Mb flanking regions of the peak SNPs, a total of 236 candidate genes were retrieved (Table S3).

**Figure 5:**
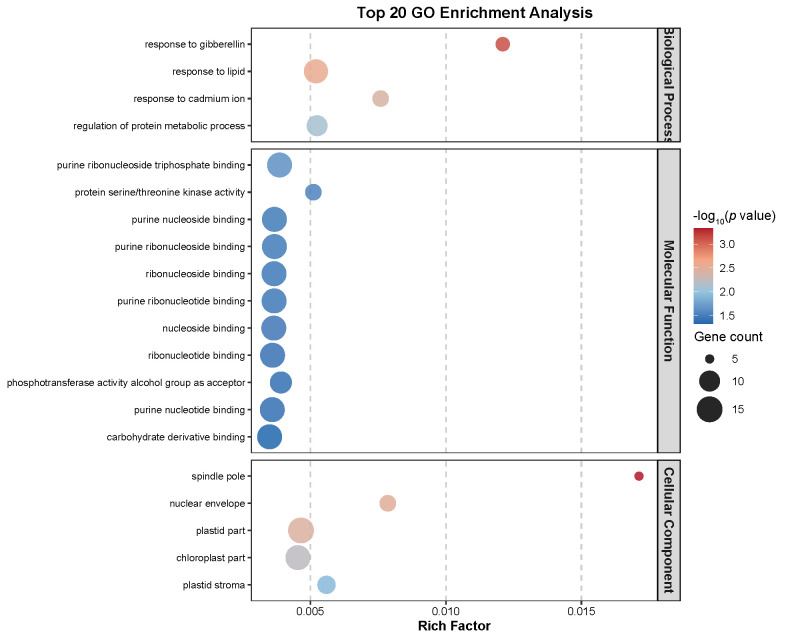
GO enrichment analysis of 236 candidate genes identified within ±0.15 Mb intervals flanking significant GWAS SNPs. Top 20 enriched GO terms in biological process (BP), cellular component (CC), and molecular function (MF) categories are shown. The colour gradient of dots corresponds to −log_10_ (*p* value) for enrichment significance, and dot size indicates the number of genes assigned to each GO term.

GO enrichment analyses were performed on the 236 candidate genes to elucidate the functional characteristics, classifying them by cellular component (CC), molecular function (MF), and biological process (BP) (Figure 5). No significantly enriched GO terms were detected at a threshold of False Discovery Rate (FDR) < 0.05. Based on the top 20 enriched GO terms, genes in the CC category were mainly distributed in GO:0000922 (spindle pole), GO:0005635 (nuclear envelope), and GO:0044435 (plastid part), with the highest number of genes assigned to plastid and chloroplast components (15 and 14, respectively), suggesting that these candidate genes are largely involved in photosynthetic organelle and basic cellular architecture. In the MF category, candidate genes were enriched in GO:0035639 (purine ribonucleoside triphosphate binding), GO:0004674 (protein serine/threonine kinase activity), GO:0032550 (purine ribonucleoside binding), and GO:0001883 (purine nucleoside binding), with nucleic acid binding and protein kinase catalytic activity as core functional features. For BP, candidate genes were primarily involved in GO:0009739 (response to gibberellin), GO:0033993 (response to lipid), and GO:0046686 (response to cadmium ion), indicating that these genes may participate in sweet corn growth, development, and stress adaptation by responding to endogenous plant hormones and external stimuli, as well as regulating protein metabolism.

## 3. Discussion

### 3.1. Phenotypic Variation

This study revealed that phenotypic variation in sweet corn agronomic traits is co-regulated by genotype and environment. Vegetative traits such as PH and EH were significantly higher in autumn than in spring, with EH showing the largest seasonal mean square (4281.54), indicating its high sensitivity to environmental cues, consistent with the plastic response of maize plants to photothermal conditions [35]. This aligns with the low light and temperature in spring and more favorable conditions in autumn in Guangdong Province, China. In contrast, TL and SD exhibited smaller seasonal differences, reflecting their phenotypic stability. Yield-related traits showed coefficients of variation ranging from 18.64% to 32.16% and high broad-sense heritability, confirming sufficient genetic diversity and phenotypic reliability for GWAS [6]. Analysis of variance further revealed that genotype effects were highly significant for all traits except tassel length, and significant genotype × season interactions were detected for ear traits, suggesting that different genotypes adopt distinct response strategies to environmental changes [36]. This finding is consistent with previous reports of environment-driven phenotypic plasticity [37]. In the context of increasing climatic variability, dissecting such interaction mechanisms is crucial for breeding stable-yielding sweet corn varieties [38]. Furthermore, the environment-dependent expression of traits with significant interactions suggests that multi-environment trials can improve selection accuracy [39]. In this study, BLUP estimates were based on only two environments (spring and autumn 2024), which may reduce the accuracy of stability assessment. Future studies with more environments are needed to validate these results.

### 3.2. Phenotypic Correlations

Understanding the correlations between plant architecture and yield traits is essential for breeding programs. We observed high genetic correlations among PH, EH, SD, and TL (Figure 2A). The high correlations likely reflect convergent selection for multiple agronomic traits during sweet corn inbred line development, resulting in pleiotropic genetic coadaptation. This result suggests a strong genetic association and the potential for a shared genetic basis [40]. In this study, the phenotypic correlation network among 11 agronomic traits exhibited significant seasonal dynamics. Under spring conditions, plant architecture traits were positively correlated with ear traits, reflecting the synchronization of vegetative and reproductive growth under favorable conditions. This is consistent with reports of significant associations between yield traits and plant structure in sweet corn [41]. However, in autumn, correlations among plant architecture traits weakened considerably. Environmental factors may trigger shifts in resource allocation strategies, leading to phenotypic decoupling among traits [42]. These results highlight the regulatory effect of environmental conditions on phenotypic correlation structures [43,44] and emphasize the need for multi-season phenotypic evaluation to avoid selection bias from a single environment. This environmental dependency of trait correlations is also observed in rice [45], where flowering time and panicle architecture correlations shift between seasons.

### 3.3. Genetic Structure

Both sNMF and DAPC analyses identified three main clusters and six subpopulations, respectively, with subpopulation classification closely corresponding to geographic origin and breeding pedigree. This structure is highly consistent with the classical framework of sweet corn ecotypes of tropical, temperate, and subtropical [46], corroborating the roles of geographic isolation and artificial selection in shaping sweet corn genetic differentiation. Similar ecotype-dependent population structures have been reported in wheat [47], where geographic isolation and breeding selection also drove differentiation into distinct genetic groups. Five admixed individuals were detected, suggesting introgression among different ecotypes during breeding, which aligns with the narrow genetic base of sweet corn and the widespread use of heterosis in breeding practice [4]. Notably, DAPC further refined the tropical × temperate hybrid subpopulation, reflecting the regional characteristics in Chinese sweet corn breeding, which can inform the precise utilization of germplasm resources [46]. The large genetic distance between tropical and temperate populations suggests that tropical germplasm could serve as a valuable resource for broadening the genetic base of temperate sweet corn [48]. These results validate the ecotype-dependent nature of sweet corn population structure.

### 3.4. LD Decay and Association Mapping

Multiple studies have reported that *LD* decay in maize occurs rapidly within 5–10 kb, with an overall decay range extending up to 300 kb and varying across chromosomes [49]. In this study, *LD* decayed to an *r*^2^ = 0.20 threshold with a distance of 0.15 Mb. Xiao et al. [33] reported an *LD* decay distance of 35 kb at in a panel of 295 sweet corn inbred lines. A decay distance of 50 kb at in a panel of 447 mixed sweet and waxy corn inbred lines were reported by Dang et al. [6]. The sweet corn inbred panel used in this study consisted of 101 elite lines, exhibiting relatively rapid *LD* decay; however, the smaller population size likely led to an overestimation of the *LD* decay distance [50]. Future studies with larger populations are needed to improve mapping resolution.

A total of 16 significant MTAs were identified in this study, associated with seven phenotypic traits. Chen et al. [51] summarized 999 QTLs for ear, kernel, and yield-related traits in maize reported between 2000 and 2016, and integrated them into 79 MQTLs. The MTAs identified in this study partially overlapped with these previously reported MQTL intervals. Specifically, eight significant SNPs were located within the conserved regions of *MQTL7*, *8*, *13*, *33*, *41*, *43* and *46* [52], confirming these genomic regions as genetic hotspots regulating FW, HW, EL, ED, ERN and KNR. In addition, SNP29364 was located within the interval of the *qKRN7-4* detected by Kong et al. [53]. Ni et al. [54] identified a candidate gene *Zm00001d002989* associated with kernel row number, encoding a cytokinin oxidase/dehydrogenase, and SNP47366 was located near this gene. This study also identified eight novel MTAs, among which SNP25518 was detected across all environments, representing a novel target for key candidate genes related to plant architecture and ear traits.

LN is a key plant architecture trait that influences canopy structure and yield potential in maize. Numerous studies have reported QTL mapping for LN. In this study, SNP27309 overlapped with the *qLA6-1* QTL interval reported by Cui et al. [55], and SNP2191 was located with the *qLN9* interval identified by Li et al. [56]. SNP53027 represents a newly identified locus, providing a novel candidate gene target for molecular genetic improvement of LN in maize. SD is an important agronomic trait reflecting stalk mechanical strength and lodging resistance. The SD-associated SNP29274 identified in this study was detected within the QTL *qSD-7* reported by Shang et al. [14], with a PVE of 15.00%, further validating the critical role of this chromosomal region in regulating stalk development.

TL directly influences tassel inflorescence architecture and pollen production. Numerous QTLs for TL and tassel branch number have been reported, distributed across all ten chromosomes except Chr 8 [57,58,59,60]. Wang et al. [61] employed QTG-Miner multi-omics analysis and cloned seven functional genes regulating tassel branch number. However, the SNP25836 locus identified in this study did not overlap with any previously reported tassel-related QTLs, representing a novel genetic locus that requires further fine mapping and validation.

This study reported five stable MTAs across environments, including a new ERN locus (SNP25518) consistently associated with ammonium transporter genes, which has not been highlighted in other sweet corn populations. These stable loci can be directly applied in marker-assisted selection, addressing a key gap in sweet corn improvement.

### 3.5. Candidate Gene Analysis

To identify putative candidate genes, we revised all genes harboring MTAs or the closest ones, and searched the confidence intervals for enriched pathways with potential roles in the expression of the studied traits. Within the ±0.15 Mb confidence interval flanking each peak SNP, 236 candidate genes were identified across the 16 significant SNP–trait associations.

Among the candidate genes associated with ear traits and yield, multiple loci harbored genes potentially involved in yield formation. For the three ear morphological traits, KNR, EL and ERN, two R2R3-MYB domain protein-encoding genes (*GRMZM2G048295* and *GRMZM5G887099*) were located within ERN-associated loci. MYB62 has been reported to regulate root architecture under low-phosphorus stress in maize [62], and transcription factors can influence ear diameter development by modulating hormone balance, cell division, and expansion [21], suggesting that these genes may integrate developmental and environmental signals during ear morphogenesis. Similar MYB transcription factors have been shown to regulate grain size and panicle architecture in rice [63], indicating a conserved role of MYB genes in cereal yield formation.

At the metabolic regulation level, two ammonium transporter 2-encoding genes within ERN loci are related to nitrogen use efficiency, playing critical roles in maize growth and productivity [64,65]. Phosphoenolpyruvate carboxylase kinase-encoding genes are associated with metabolic regulation and may participate in carbon assimilation during the grain-filling period [66]. In addition, a pentatricopeptide repeat protein-encoding gene, *GRMZM2G332843*, within an ERN locus, may be involved in maize kernel development and starch quality [67,68]. PPR proteins are also reported to influence grain filling and starch accumulation in rice [69].

Among loci associated with plant architecture traits, *GRMZM2G009506* is a member of the *liguleless* gene family, which are classic regulators of leaf development and plant architecture in maize and have been identified as key genes controlling domestication-related traits [70]. Similar *liguleless* orthologs have been shown to regulate leaf angle and plant architecture in rice [71], suggesting a conserved role in cereal domestication and canopy architecture. For LN, several candidate genes involved in hormone signaling were identified. A serine/threonine protein kinase SRK2A-encoding gene is a core component of the ABA signaling pathway, which plays an important role in maize growth [72]. A Whirly1 protein-encoding gene is involved in maintaining chloroplast genome stability, while RCC1 domain-containing proteins have been demonstrated to be involved in plant growth and abiotic stress responses [73]. A BTB/POZ-MATH domain protein-encoding gene often functions as a substrate adaptor for ubiquitin ligases in stress signal transduction [73].

The panel size in this study is smaller than many previous maize GWAS. This may limit detection power and inflate LD decay estimates. In addition, our candidate gene identification was based solely on prediction without functional validation. Future studies should validate these candidates in larger populations and through transcriptomic or knockout approaches.

## 4. Materials and Methods

### 4.1. Plant Materials

A core germplasm collection comprising 101 elite sweet corn (*Zea mays* L. var. *saccharata*) inbred lines, developed by the fresh corn research group at the college of agriculture, South China Agricultural University, was used in this study.

### 4.2. Phenotypic Data Collection

Field experiments were conducted during two growing seasons (spring and autumn in 2024) at the teaching and research base of South China Agricultural University, Zengcheng, Guangzhou (23°14′ N, 113°38′ E). Sowing was performed in late February for the spring season and in late August for the autumn season. A randomized complete block design with three replications was adopted. Each inbred line was planted in two-row plots with 20 plants per plot, spaced 30 cm within rows and arranged in alternating wide (80 cm) and narrow (60 cm) row spacings. Fertilization and field management followed standard local practices. At the milk stage, when vegetative growth had ceased and reproductive development had stabilized, five consecutive plants from each replication were sampled for plant architecture and ear trait evaluation.

Plant architecture traits included PH, EH, SD, LN, and TL. PH was measured from the base of the stem at ground level to the tip of the tassel. EH was measured from the ground surface to the node bearing the primary ear. SD was measured at the middle of the third internode above ground using a digital caliper. LN was determined by counting all fully expanded functional leaves above the primary ear node. TL was measured from the base of the tassel to the tip of the main axis using a ruler.

Ear traits comprised FW, HW, EL, ED, ERN, and KNR. At the milky stage, intact ears were harvested. FW was measured before removing husks; HW was recorded after complete husk removal. EL was measured from the base to the tip of the ear. ED was measured at the midpoint of the ear. ERN was determined by counting the total number of kernel rows around the ear circumference at the middle section. KNR was recorded as the number of fully developed kernels in one complete row at the ear middle section.

In Guangzhou, during the 2024 growing seasons, the spring period (March–May) recorded average temperatures of 16–31 °C, total precipitation of 669 mm across 42 rainy days, and consistently high relative humidity exceeding 80%. In contrast, the autumn season (September–November) experienced average temperatures of 19–32 °C, only 187 mm of rainfall over 16 rainy days, and lower relative humidity of approximately 60%. The spring and autumn seasons exhibited marked climatic differences.

### 4.3. SNP Genotyping

Genomic DNA extraction and SNP genotyping for the 101 sweet corn inbred lines were performed by China Golden Marker Biotech Co., Ltd. (Beijing, China). Fresh leaf tissue (100 mg) was collected from 10-day-old seedlings of each line, and genomic DNA was extracted using the CTAB method. DNA integrity and purity were assessed by 1% agarose gel electrophoresis, and DNA concentration was measured using a spectrophotometer. Only DNA samples with clear bands, no degradation, and no detectable protein or RNA contamination were used for subsequent SNP genotyping.

Qualified DNA samples were genotyped using the MaizeSNP50 Genotyping BeadChip (Illumina, San Diego, CA, USA), which contains 50,000 high-quality maize-specific SNP markers evenly distributed across all 10 chromosomes, providing sufficient marker density for GWAS in the sweet corn population. Genotype calling, clustering, and initial quality control were conducted using Illumina’s GenomeStudio v2.0 software (Illumina, San Diego, CA, USA).

SNP marker positions were aligned and annotated against the maize B73 reference genome (B73 RefGen_v3). Physical positions were obtained by aligning all probe sequences to the reference genome, and genetic positions were corrected using published high-density genetic linkage maps of maize. The resulting chromosome number, physical position, and genetic position for each qualified SNP formed the basis for subsequent population structure analysis, linkage disequilibrium analysis, and genome-wide association study.

### 4.4. Statistical Analysis

Best linear unbiased predictions (BLUPs) for all traits were estimated separately for each growing season (1) and across both seasons (2) using the following models:

(1) $$y=\mu +g_{i}+r_{j}+\varepsilon_{ij}$$

(2) $$y=\mu +g_{i}+r_{j}+s_{k}+{\left(gs\right)}_{ik}+\varepsilon_{ijk}$$

where μ is the overall mean, $g_{i}$ is the random effect of the *i*th genotype, $r_{j}$ is the random effect of the *j*th replicate, $s_{k}$ is the random effect of the *k*th growing season, ${\left(gs\right)}_{ik}$ is the random effect of the interaction between the *i*th genotype and the *k*th season, and $\varepsilon_{ijk}$ is the random error. All predictors were treated as random effects. Models were fitted using the *lmer* function in the R package *lme4* v1.1-35 [74]. BLUP values extracted from both models were used to estimate trait correlations and to perform GWAS for each season and across seasons. Analysis of variance (ANOVA) was used to test the main effects of genotype, season, and their interaction. Phenotypic correlations between traits across the two seasons were computed using the R package corrplot v0.92.

Raw SNP data were filtered to remove markers with missing chromosome or physical position information, as well as duplicate probes at the same genomic physical position. Using PLINK v1.9 [75], we retained high-quality SNPs with a missing rate ≤ 5% and minor allele frequency (MAF) ≥ 0.05 for subsequent analyses. Population genetic structure was inferred using the R packages *LEA* v3.0 [76] and DAPC v2.1 [77]. SNP data in high linkage disequilibrium (*LD*, (*r*^2^ > 0.2)) were filtered using PLINK 1.9 [75]. The resulting genotype data were converted to the geno format, and the sparse non-negative matrix factorization (sNMF) algorithm [76] was applied for population structure analysis. The number of ancestral clusters K was tested from 2 to 10, with 10 independent runs per K. The optimal K was selected based on the cross-entropy criterion.

*LD* among markers was estimated using TASSEL v5.2.87 [78]. The decay of *LD* with physical distance was calculated following the method described by Remington et al. [79]. The threshold of *r*^2^ = 0.20 was chosen as a common standard for LD decay in maize GWAS, effectively balancing linkage block resolution and statistical power [80], providing a candidate gene search window for subsequent analyses. The genomic relationship matrix (GRM) was calculated using GCTA v1.94.1 [81] and used as a covariate in GWAS.

Genome-wide association analysis was performed using the R package GAPIT v4.0 [82], employing the FarmCPU model, which balances false-positive control and detection power for small-effect loci [83]. In total, 30,597 high-quality SNP markers after quality control were used. FarmCPU was performed with the first three principal components as fixed covariates to control population structure, and GRM was incorporated as a random effect within the model. The Bonferroni-corrected genome-wide significance threshold was set as the reciprocal of the total number of SNPs, corresponding to *p* = 3.26 × 10^−5^ [84]. Candidate gene regions were defined based on the population *LD* decay distance flanking the significant SNPs. Annotated genes within the target intervals were retrieved from the MaizeGDB database [85], and Gene Ontology (GO) enrichment analysis was performed using agriGO v2.0 [86].

## 5. Conclusions

In this study, GWAS using the FarmCPU model was conducted across two growing seasons on 11 agronomic traits in a panel of 101 sweet corn elite lines. Combining sNMF and DAPC stratified germplasm into three broad ancestral clusters and six geographically distinct subpopulations, while FarmCPU identified 16 MTAs for seven traits, five of which were stable across seasons with 8.0–16.3% PVE. The single most important finding is five environment-consistent major loci governing kernel row number, kernel number per row and leaf number above ear, including the universal stable SNP25518 unique to sweet corn germplasm, supplying reliable markers for yield improvement. For sweet corn breeding, tropical–temperate inter-subpopulation crossing can expand narrow genetic diversity, and the stable SNPs can be developed into key markers for the marker-assisted selection of high-yield lines. Recommended next steps include fine-mapping novel loci, validating candidate gene functions via transformation, and integrating major MTAs into genomic prediction to accelerate breeding cycles. Limitations include a small panel of 101 lines, single-site phenotyping without multi-region validation, and lack of gene functional verification. Overall, the MTAs and candidate genes identified in this study provide a valuable foundation for high-yield sweet corn molecular breeding.

## Figures and Tables

**Table 1:** Variance analysis of 11 agronomic traits in sweet corn.

Trait	Item	Sum of Square	df	Mean Square	*F*-Value
PH	Gen.	90,993.93	100	909.94	1.89 **
	Season	2186.90	1	2186.90	4.54 *
	Gen. × Season	29,625.54	100	296.26	0.62
EH	Gen.	18,868.56	100	188.69	1.82 **
	Season	4281.54	1	4281.54	41.36 **
	Gen. × Season	3473.23	100	34.73	0.34
SD	Gen.	678.73	100	6.79	1.63 **
	Season	35.81	1	35.81	8.58 **
	Gen. × Season	277.09	100	2.77	0.66
LN	Gen.	120.34	100	1.20	5.98 **
	Season	1.20	1	1.20	5.98 *
	Gen. × Season	49.30	100	0.49	2.45 **
TL	Gen.	1779.54	100	17.80	1.27
	Season	2.26	1	2.26	0.16
	Gen. × Season	1030.06	100	10.30	0.73
FW	Gen.	1,838,209.99	100	18,382.10	12.23 **
	Season	134.96	1	134.96	0.09
	Gen. × Season	251,853.44	100	2518.53	1.68 **
HW	Gen.	807,032.08	100	8070.32	19.70 **
	Season	762.80	1	762.80	1.86
	Gen. × Season	76,325.57	100	763.26	1.86 **
EL	Gen.	5388.11	100	53.88	4.18 **
	Season	19.02	1	19.02	1.47
	Gen. × Season	1693.03	100	16.93	1.31 *
ED	Gen.	10,785.84	100	107.86	10.90 **
	Season	15.54	1	15.54	1.57
	Gen. × Season	2475.53	100	24.76	2.50 **
EN	Gen.	2543.96	100	25.44	16.90 **
	Season	2.91	1	2.91	1.93
	Gen. × Season	505.76	100	5.06	3.36 **
KNR	Gen.	14,174.25	100	141.74	36.86 **
	Season	0.33	1	0.33	0.09
	Gen. × Season	2981.59	100	29.82	7.75 **

Note: * and ** represents significance at the 0.05 and 0.01 level, respectively. Gen. represents the genotype.

**Table 2:** GWAS-based identified SNPs in spring, autumn, and 2Ssns.

Season	Trait	SNP	Chr	Alleles	Pos	−log_10_ (*p*)	MAF	Effect *	*R* ^2^
Spring	LN	SNP53027	6	A/G	150,667,306	4.75	0.47	−0.20	11.98%
		SNP27309	6	A/G	71,921,054	4.67	0.45	−0.20	12.24%
	FW	SNP2943	5	A/G	60,002,641	4.67	0.37	25.60	11.68%
		SNP2942	5	A/G	60,002,820	4.67	0.37	25.60	11.68%
		SNP53698	5	A/G	60,032,222	4.67	0.37	25.60	11.99%
	EL	SNP48185	1	A/G	196,427,247	4.52	0.06	−1.75	8.03%
	ERN	SNP29364	7	A/G	19,055,024	4.65	0.35	−1.02	10.88%
		SNP25518	5	A/G	186,506,816	4.59	0.07	−1.69	10.22%
	KNR	SNP3775	1	A/G	2,045,583	5.27	0.31	−2.69	12.37%
	KNR	SNP41368	1	A/G	1,957,167	5.16	0.30	−2.71	11.95%
Autumn	SD	SNP29274	7	A/G	14,886,758	4.49	0.43	0.21	15.11%
	TL	SNP25836	5	A/G	201,866,173	4.63	0.36	−0.01	11.82%
	ERN	SNP25518	5	A/G	186,506,816	4.62	0.07	−1.72	9.82%
		SNP47366	2	A/C	30,261,576	4.60	0.40	−1.01	12.33%
	KNR	SNP3770	1	A/C	2,043,319	4.83	0.46	2.66	14.40%
2Ssns	LN	SNP2191	9	A/G	110,657,630	4.73	0.42	0.13	11.10%
	ERN	SNP25518	5	A/G	186,506,816	5.37	0.07	−1.51	10.53%
		SNP29364	7	A/G	19,055,024	5.30	0.35	−0.89	12.53%
		SNP25505	5	A/C	185,781,516	4.98	0.08	−1.44	10.05%
	KNR	SNP3770	1	A/C	2,043,319	5.32	0.46	2.15	16.28%
		SNP3775	1	A/G	2,045,583	5.10	0.31	−1.94	11.20%
		SNP41368	1	A/G	1,957,167	4.78	0.30	−1.91	9.74%

Note: * The effects of the MTAs are reported in respect of the effect of the rare allele of each locus; *R*^2^, percentage of phenotypic variation explained by the MTA.

## Data Availability

Data are contained within this article or Supplementary Materials.

## Supplementary Materials

The following supporting information can be downloaded at https://www.mdpi.com/article/10.3390/plants15152406/s1: Figure S1: Genome-wide SNP density distribution across 10 chromosomes; Figure S2: Manhattan plots and QQplots; Table S1: Descriptive statistics of the studied traits; Table S2: List of genotypes, their origin and genetic structure; Table S3: Candidate genes identified within ±0.15 Mb intervals flanking significant GWAS SNPs across 11 agronomic traits in sweet corn. Red fonts dedicate the 15 candidate genes at the peak SNPs.
