# Supplementary material for: Genome-Wide Association Studies of Agronomic and Yield Traits in Sweet Corn (Zea mays L. var. saccharata)
Source: Plants (Basel). 2026 Aug 6;15(15):2406. doi: 10.3390/plants15152406 (PMC13468538; doi:10.3390/plants15152406)
Supplement: Supplementary file 1 [file plants-15-02406-s001.zip › Figure S1.pdf]

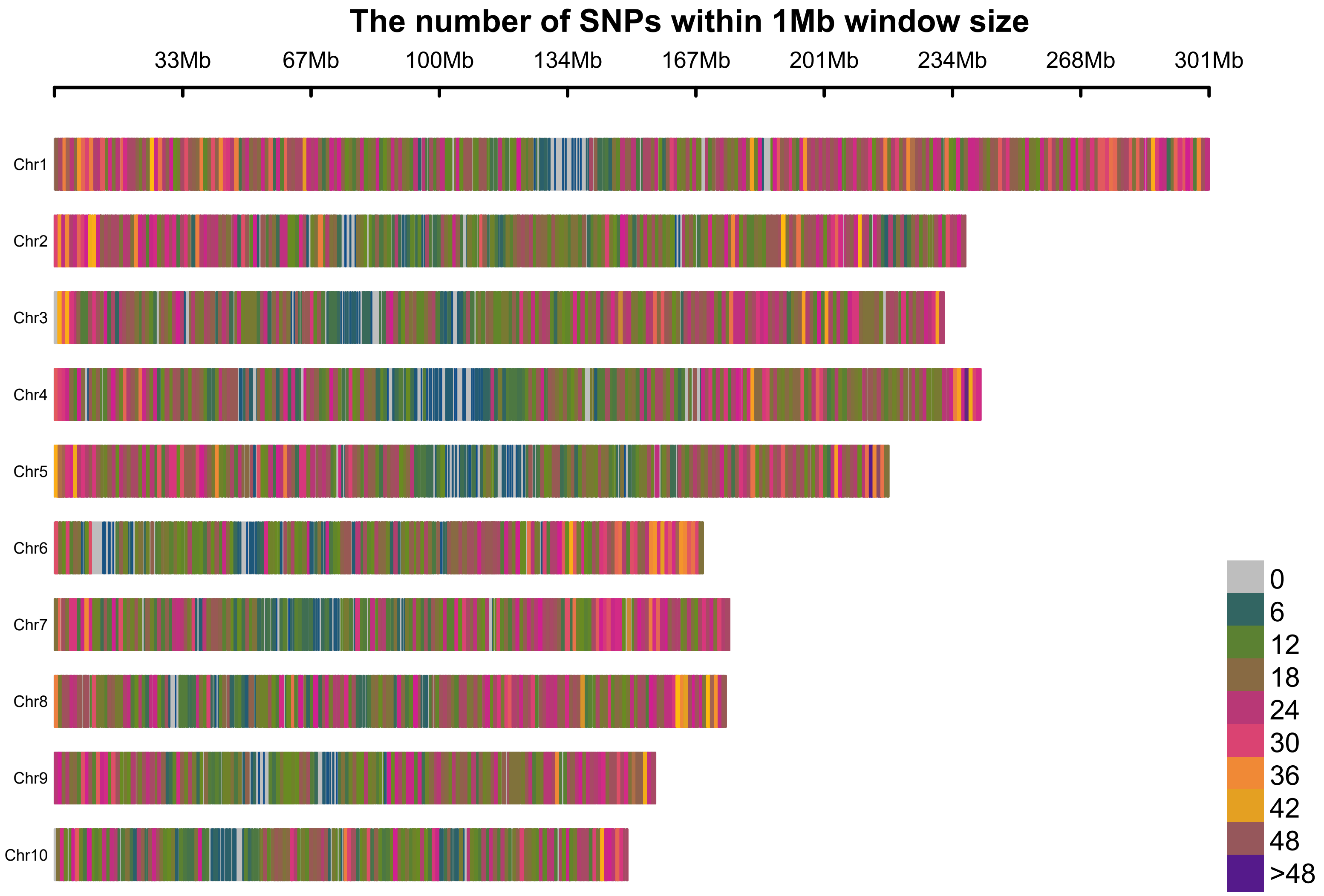
The number of SNPs within 1Mb window size
33Mb
67Mb
100Mb
134Mb
167Mb
201Mb
234Mb
268Mb
301Mb
Chr1
Chr2
Chr3
Chr4
Chr5
Chr6
Chr7
Chr8
Chr9
Chr10
0
6
12
18
24
30
36
42
48
>48
