# Supplementary material for: Genome-Wide Association Studies of Agronomic and Yield Traits in Sweet Corn (Zea mays L. var. saccharata)
Source: Plants (Basel). 2026 Aug 6;15(15):2406. doi: 10.3390/plants15152406 (PMC13468538; doi:10.3390/plants15152406)

Spring_LN

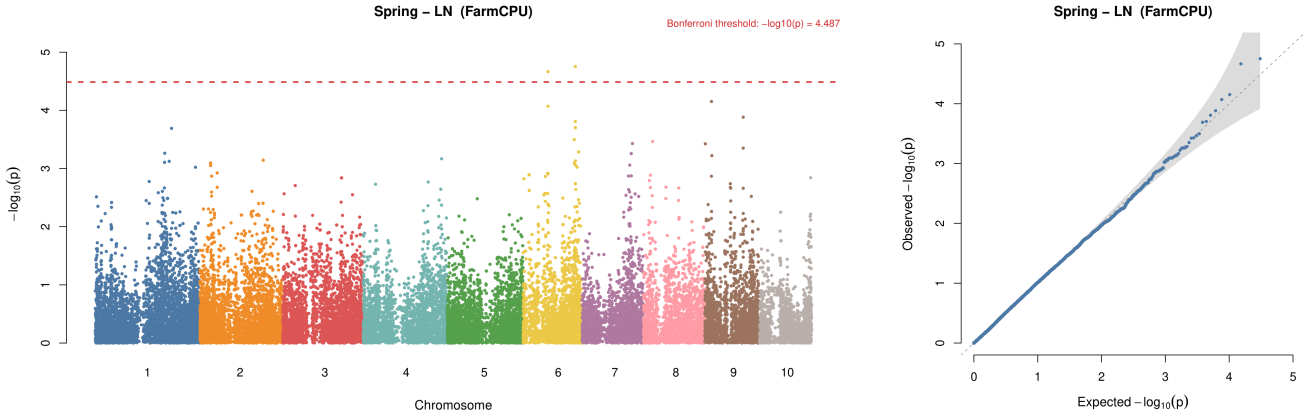

Spring_FW

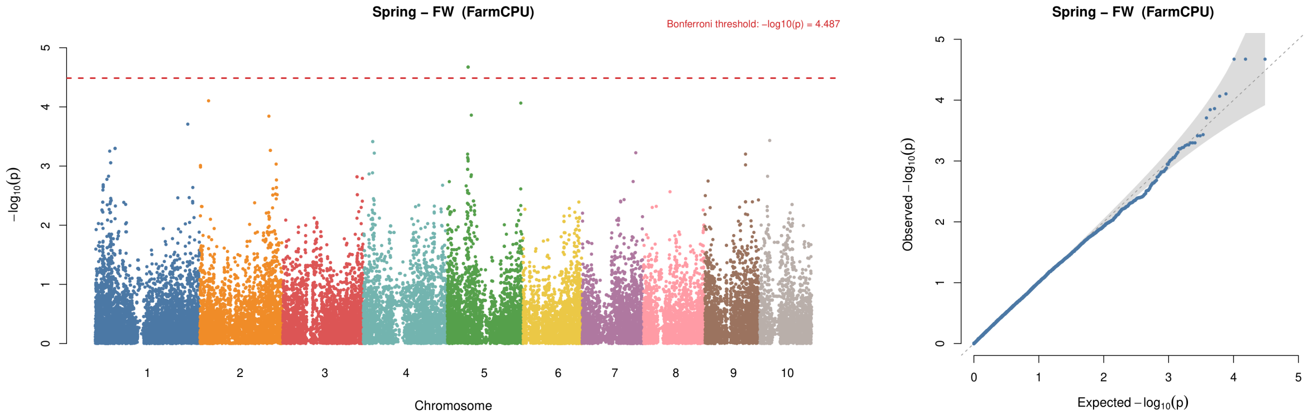

Spring_EL

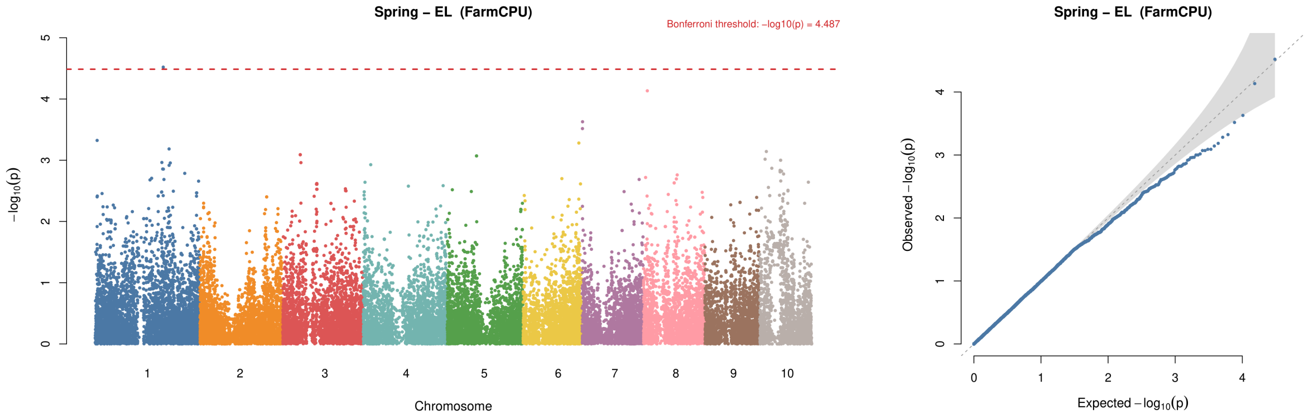

Spring_ER

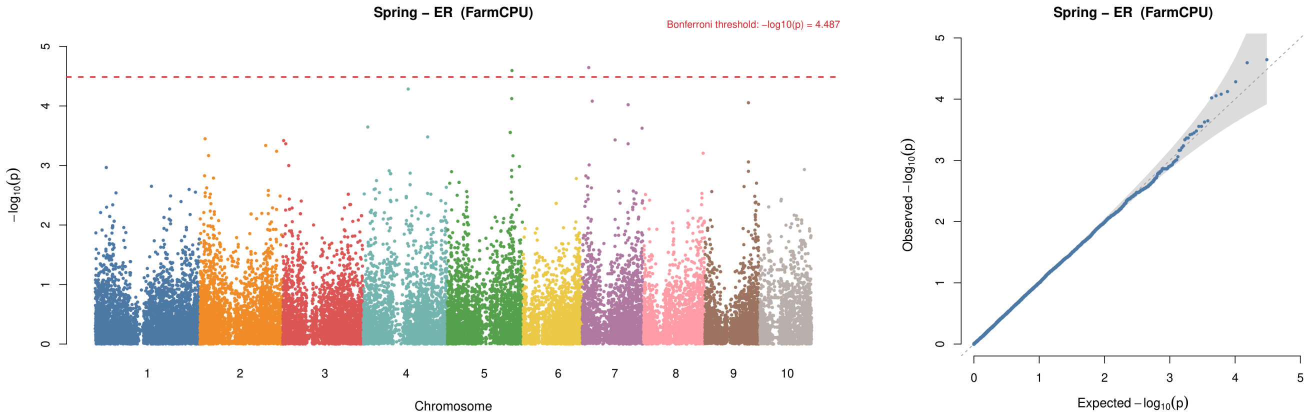

Spring_RN

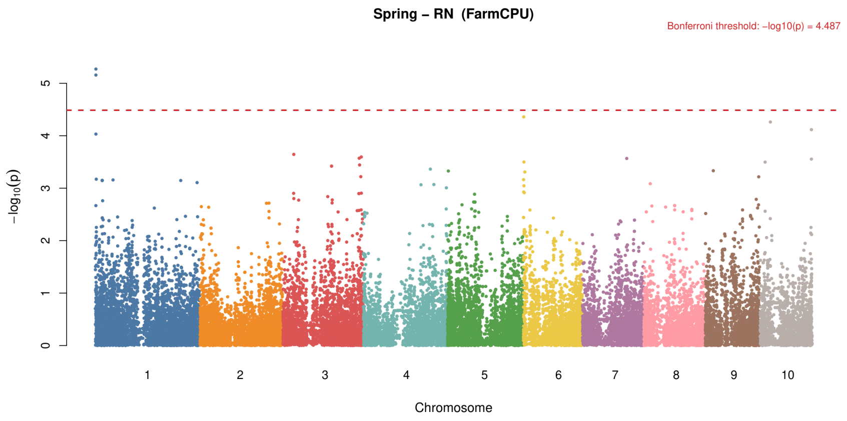

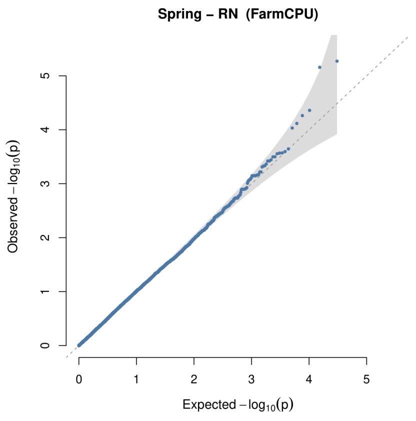

Autumn_SD

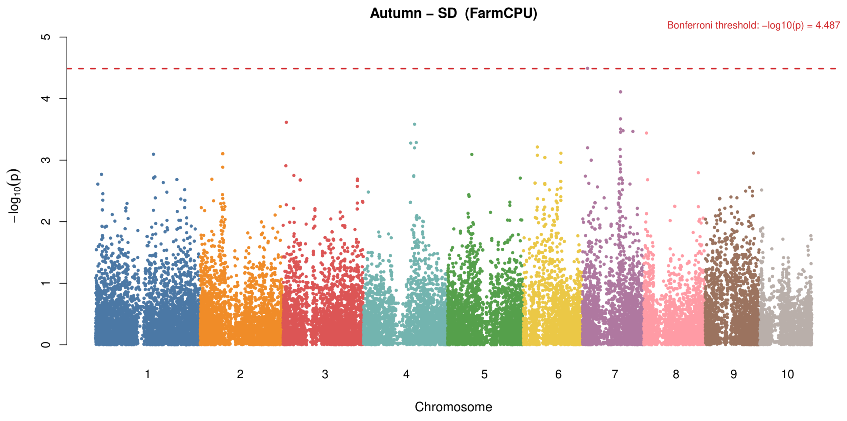

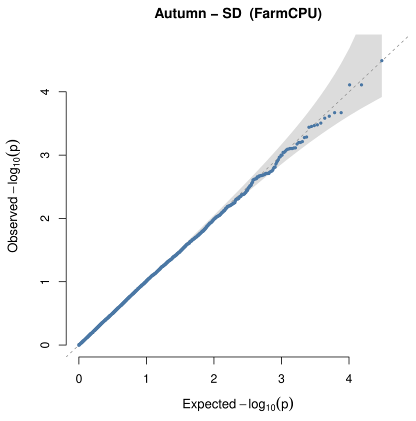

Autumn_SL

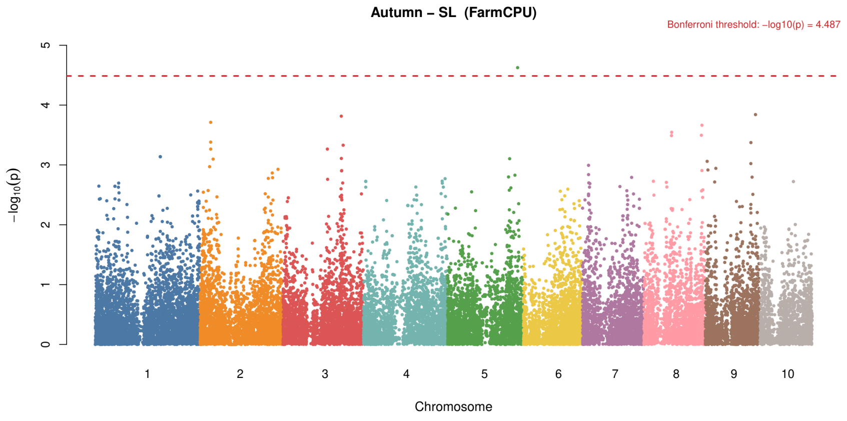

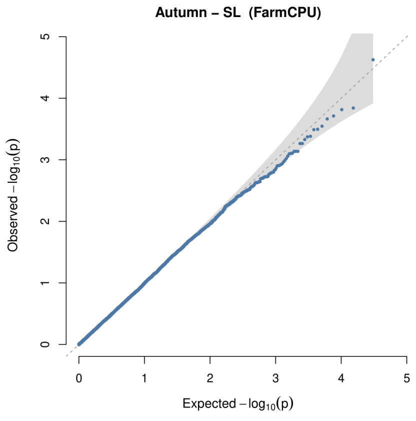

Autumn_ER

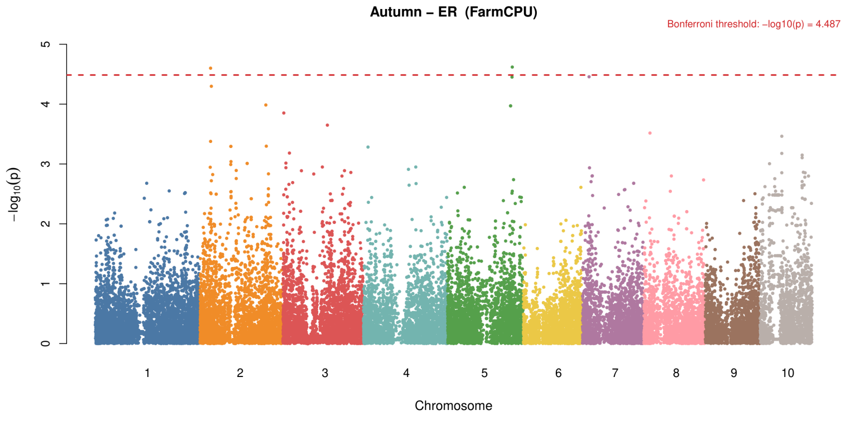

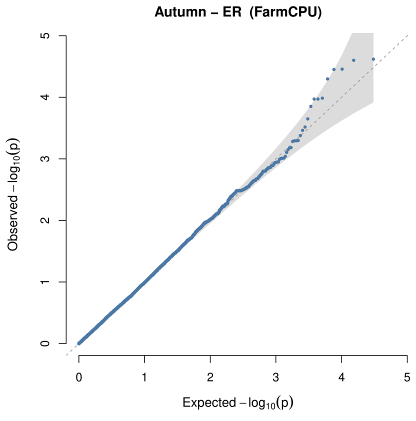

Autumn_RN

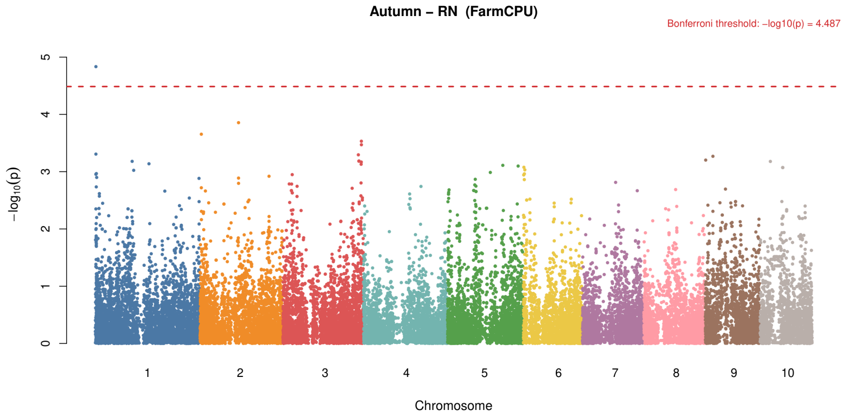

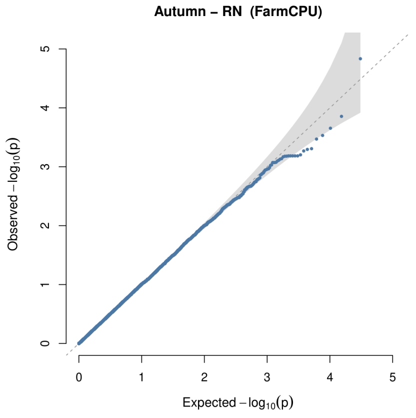

coBLUP_LN

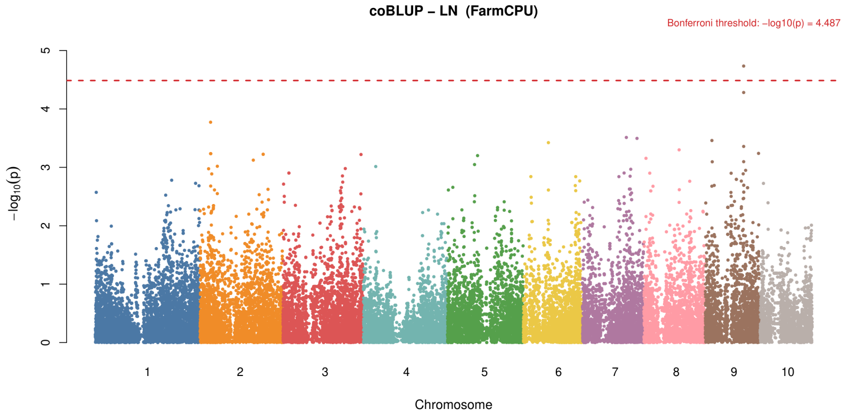

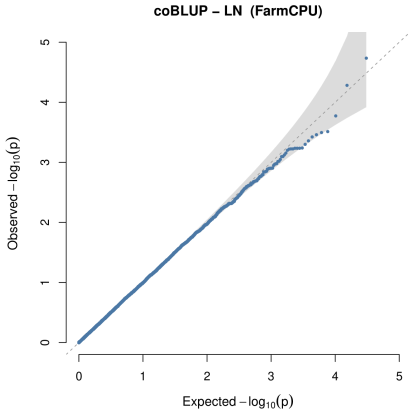

coBLUP_ER

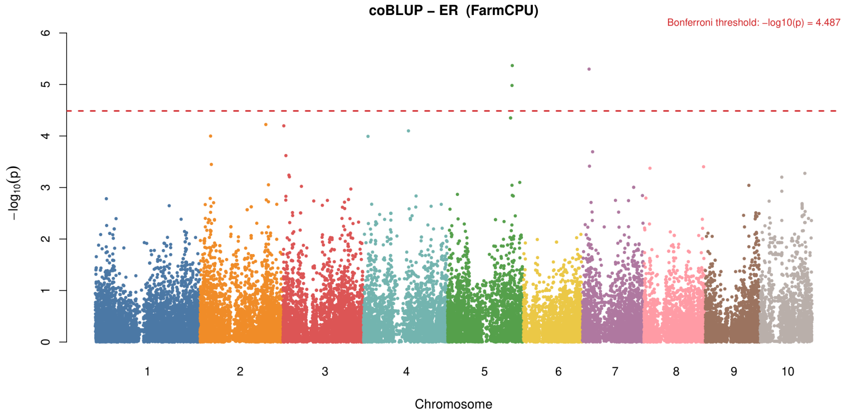

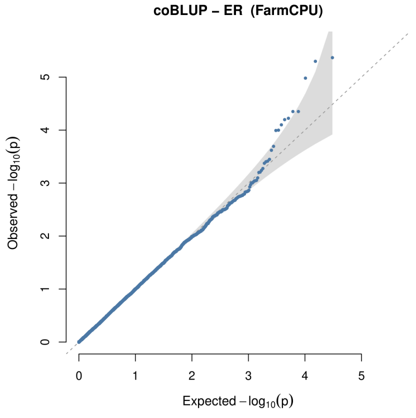

coBLUP_RN

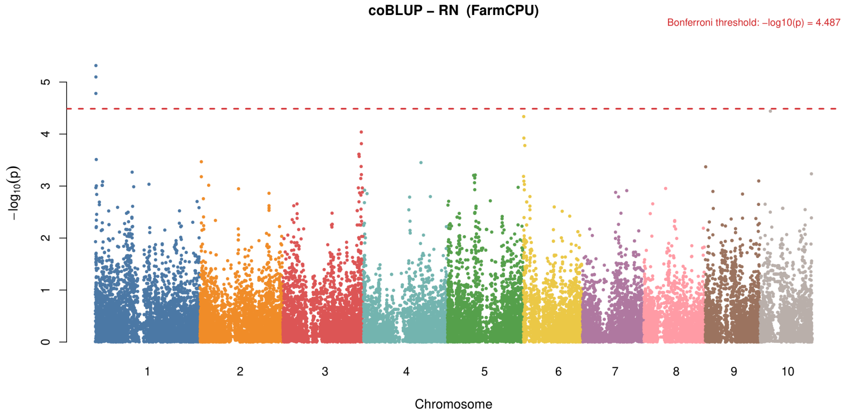

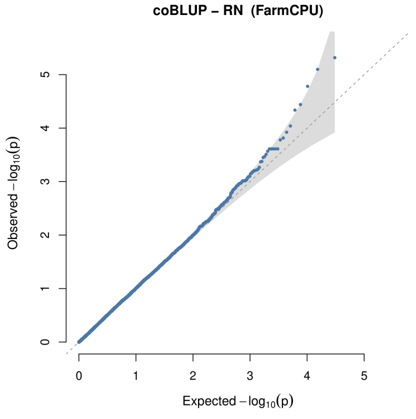

Supplement: Supplementary file 1 [file plants-15-02406-s001.zip › Figure S2.pdf]
